# Development of the Social Network-Based Intervention “*Powerful Together with Diabetes*” Using Intervention Mapping

**DOI:** 10.3389/fpubh.2017.00334

**Published:** 2017-12-18

**Authors:** Charlotte Vissenberg, Vera Nierkens, Paul J. M. Uitewaal, Barend J. C. Middelkoop, Giel Nijpels, Karien Stronks

**Affiliations:** ^1^Academic Medical Center (AMC), Amsterdam, Netherlands; ^2^The Hague’s Public Health Department, The Hague, Netherlands; ^3^Leiden University Medical Center, Leiden, Netherlands; ^4^VU University Medical Center, Amsterdam, Netherlands

**Keywords:** diabetes self-management, deprived neighborhoods, type 2 diabetes, social network, social influences and social support

## Abstract

This article describes the development of the social network-based intervention *Powerful Together with Diabetes* which aims to improve diabetes self-management (DSM) among patients with type 2 diabetes living in socioeconomically deprived neighborhoods by stimulating social support for DSM and diminishing social influences hindering DSM (e.g., peer pressure and social norms). The intervention was specifically developed for patients with Dutch, Turkish, Moroccan, and Surinamese backgrounds. The intervention was developed according to Intervention Mapping. This article describes the first four steps of Intervention Mapping: (1) the needs assessment; (2) development of performance and change objectives; (3) selection of theory-based methods and strategies; and (4) the translation of these into an organized program. These four steps resulted in *Powerful Together with Diabetes*, a 10-month group-based intervention consisting of 24 meetings, 6 meetings for significant others, and 2 meetings for participants and their spouses. The IM method resulted in a tailored approach with a specific focus on the social networks of its participants. This article concludes that the IM method helped our planning team to tailor the intervention to the needs of our target population and facilitated our evaluation design. However, in hindsight, the intervention could have been improved by investing more in participatory planning and community involvement.

## Introduction

People in lower socioeconomic groups, including ethnic minorities, are not only disproportionately affected by type 2 diabetes, they also have more diabetes-related complications and higher diabetes-related mortality compared with patients in higher socioeconomic groups (1–3). A recent study among ethnic minorities in the Netherlands shows that of those patients that were medically treated, only 37–53% had HbA1c levels on target (4). To the best of our knowledge, no comparable information on glycemic control in Dutch patients in lower socioeconomic groups in the Netherlands is known.

A poorer glycemic control, related to less adequate self-management behaviors, partly accounts for these increased risks (5). Properly managing type 2 diabetes requires a schedule of extensive self-management behaviors. These include an adequate use of medications, if applicable self-monitoring of blood glucose, eating healthy and being physical active, regularly checking and taking adequate care of the feet and dealing adequately with diabetes in every situation (6). Complying with and maintaining such complex health regimens seem to be challenging, especially for socioeconomically deprived patients (7–9).

Low incomes, a low educational level and living in a deprived neighborhood are associated with a lower prevalence of blood glucose monitoring and not knowing how to deal with extreme blood glucose levels (10, 11). Furthermore, especially the combination of smoking, physical inactivity and a lower consumption of fruit and vegetables is prevalent in socioeconomically deprived groups in the Netherlands (12–16). There is thus a need for effective interventions that improve glycemic control among socioeconomically deprived patients with type 2 diabetes.

There are multiple factors that influence self-management behaviors among socioeconomically deprived patients, such as a lack of knowledge, low health literacy, low risk perception, low outcome expectations, low self-efficacy, and specific socioeconomic barriers (lack of financial resources and environmental factors) (7, 9, 17–21). However, an increasing amount of studies shows that social interactions with friends and family members have a major impact on self-management behaviors as well.

Social support, the aid and assistance exchanged through social relationships and interpersonal transactions, can positively influence self-management, but significant others can also hinder self-management by interfering with or paying too much attention to self-management (22, 23). Socioeconomically deprived patients seem to have less access to supportive social networks, to generally have fewer sources of social support in their social environments and to receive less social support, which is needed for adherence (24–26). In addition, they are often confronted with social influences from their immediate social environments that hinder self-management (e.g., peer pressure, specific cultural beliefs and expectations, and fewer positive role models) (27, 28). Interventions that target social influences affecting self-management behavior, such as social support, might be promising. To the best of our knowledge, there are no interventions that focus simultaneously on social support and hindering social influences in this target population.

Therefore, we developed a social network-based intervention (*Powerful Together with Diabetes*) that aimed to stimulate social support for self-management and to diminish hindering social influences on diabetes self-management (DSM) among socioeconomically deprived patients. This target population often has poor reading and writing skills (20). Furthermore, using electronical devices and participating in online communities might be hindered by a low income and low health literacy (29, 30). Therefore, in our study design, we choose to focus on real-life social networks instead of focusing on online social networks.

We used IM to develop *Powerful Together with Diabetes* (31). The development was part of a 4-year project consisting of the development, implementation and evaluation of the intervention. We had 1 year for the intervention development and preparation for implementation. The study design, results of the process evaluation, and the effects of the intervention on outcome measures are described in a number of papers (32–34).

This article describes the first four steps of the intervention development. The development of the entire intervention is described in detail in the handbook and the materials, which are available from the author. The development of the most distinctive features of this intervention is outlined here including the specific focus on social support and social influences that affect self-management, particularly in patients from socioeconomically disadvantaged neighborhoods, and on examining the specific educational requirements of patients living in such neighborhoods.

## Materials and Methods

The first four phases of Intervention Mapping consist of the following: (1) conducting a needs assessment, (2) creating performance and change objectives, (3) selecting theory-based intervention methods and practical strategies, and (4) translating methods and strategies in an appropriately organized program. This section describes the methods used for the needs assessment.

### Needs Assessment

The needs assessment consisted of a literature review and a qualitative study. The most current version of the IM handbook specifically focuses on participatory planning (35). However, at the beginning of this project in the IM handbook of 2006, this focus was less extensive (31). We encountered some barriers to participatory planning as advocated by the IM method. For example, we did not know the exact neighborhoods in which the intervention would be implemented due to difficulties with the recruitment of general practitioners (GPs). We planned to aim the intervention at a very specific target population (with suboptimal glycemic control) and did not want to create false expectations within a community. Therefore, we did not precisely know which community to address. Because of these barriers combined with time constraints, we choose to focus more on other aspects of the intervention development instead of on participatory planning.

#### Literature Review

The needs assessment started with a scoping review of the literature (unpublished) on social support and an exploration of theories related to self-management: the theory of self-regulation, different self-management theories, and the transactional model of stress and coping, relapse prevention, and social learning theories (36–40). The databases PubMed, Embase, PsycINFO, and Google Scholar were searched for articles describing the influence of social support on diabetes outcomes and DSM. A search was also made for intervention studies that aimed to increase or create social support for DSM.

The literature review provided a general overview of the most important factors related to self-management of people with type 2 diabetes, i.e., low outcome expectations, low self-efficacy, lack of knowledge on diabetes, and low risk perception (7, 9, 17–21). The review also revealed that not only social support but also other social influences in the immediate social environments of the patients (e.g., peer pressure, social norms, and role models) are important for self-management, especially in patients from socioeconomically deprived neighborhoods.

However, information was lacking on the precise nature of these determinants among our target population. For example, which social norms and role models are present in the social environment of these patients and how do these affect self-management behaviors? What form does peer pressure take and how is this related to self-management behaviors? How can these determinants be influenced? With the aim to address these questions, we then conducted a qualitative study to further examine the relation between social support and other social determinants on self-management behaviors. This qualitative study was conducted among people with type 2 diabetes from socioeconomically deprived neighborhoods, particularly Turkish, Moroccan, Surinamese, and Dutch patients.

#### Qualitative Study

During this project, we had 1 year for the intervention development and the preparation for implementation (recruitment of GPs and patients, writing our proposal for the medical ethical committee). In practice, we had 4 months for our needs assessment.

The qualitative study consisted of semi-structured in-depth interviews with health-care professionals and their patients. In addition, participant observations took place and we analyzed a forum held for people with diabetes. Because of time constraints, we decided that 24 in-depth interviews would be attainable. To further validate our findings, we also re-analyzed the in-depth interviews and focus group discussions with people with diabetes that were organized and conducted by fellow researchers.

##### Interviews

Semi-structured in-depth interviews were held with people with diabetes from lower socioeconomic groups (*n* = 21) and their health-care professionals (*n* = 3). They were recruited *via* a diabetes nurse who was working in a socioeconomically deprived neighborhood and *via* an advertisement placed on a diabetes forum. All three health-care professionals (two diabetes nurses and one general practice assistant) worked in socioeconomically deprived neighborhoods and were recruited with the help of GPs involved in this study.

The 21 patient interviews were conducted at the respondents’ homes or, if preferred, at a local community center or health-care center (each interview lasted 60–90 min). For all interviews, a topic list was used which was revised in the light of emerging findings. Relevant topics (Table 1) included as follows: self-management in daily life, barriers and facilitators to self-management, interactions with health-care professionals, and the role of relatives/friends in self-management.

**Table 1:** Topic list for patients from a socioeconomically deprived neighborhood.

**1. Living with diabetes**
– When were you diagnosed with diabetes? – What do you have to do to keep your diabetes under control on a daily basis? – How did your life change since the diagnosis? – What did you find difficult to change? What did you find the most difficult to get used to? Do you still experience difficulties with certain aspects of diabetes self-management (DSM)? Which aspects? Why?
**2. Self-management**
*Medications* – Which medications do you take? When do you take these? – How do you fit your medication use into your daily life? Do you find that difficult? – Do you always manage to take your medications correctly and on time? – Could you specify a situation in which you did *not* manage to take your medications? How do you deal with these situations? – What do you need to take your medications correctly and on time (practical and social support)? What could your significant others have best done in this situation?
*Insulin* – Do you use insulin? When? – How do you fit your insulin use into your daily life? Do you find that difficult? – Do you always manage to take your insulin correctly and on time? – Can you specify a situation in which you did not manage to take your insulin? How do you deal with these situations? – What do you need to take your insulin correctly and on time (practical and social support)? What can your significant others do for you in these situations?
*Nutrition* – In what way(s) did you adapt your nutrition when you were diagnosed with diabetes? Did you find that difficult? Do you still find that difficult? – Do you manage to eat sufficient healthy foods every day? Do you manage to eat regularly every day? Do you manage *not* to eat too many calories every day? – Can you name some obstacles/situations in which it is difficult to manage healthy eating? (e.g., holidays, busy schedules, and bad mood) – How do you deal with situations like these? In these situations, what do you need to be able to eat healthily? What can your significant others do for you in these situations?
*Physical activity* – Do you exercise more since the diagnosis? How do you experience this? – Do you manage to get enough exercise every day? – Can you describe obstacles/situations in which it is difficult to exercise enough? (e.g., holidays, busy schedules, and bad mood) – How do you deal with situations like these? What do you need to get enough exercise in these situations? What can your significant others do for you in these situations?
*Smoking* – Do you smoke? – Can you describe obstacles/situations that make it difficult for you to quit smoking? – How do you deal with situations like these? What do you need so that you will not start smoking in these situations? What can your significant others do for you in these situations? – Former smoker: are you ever tempted to start smoking again? In which situations? What do you need so that you will not start smoking in these situations? What can your significant others do to support you?
**3. Role of significant others (family members, friends, acquaintances)**
Which people are important for your DSM? What is their role in your DSM? Do they help or support you? With what? How? How do you experience that? For your DSM, what kind of support do you receive that you really appreciate? With regard to your self-management, what do you appreciate most about your family members and significant others? What is it that these people do that makes them supportive of your self-management? What can they do that you find difficult? How do they support you? Are you sometimes confronted with beliefs about diabetes that are incorrect? How do you deal with these beliefs? How do these beliefs affect you? Do you behave differently because of these beliefs? Do you ever experience difficulties in managing your diabetes when you are with others? Could you describe such a situation? What happens in these situations? How do you feel in these situations? What do you need in these situations? Does it ever happen that people in your immediate social environment do not take your DSM into account? Can you describe such a situation? What happens in these situations? How do you feel in these situations? What do you need in these situations? Does it ever happen that family members or friends make it difficult for you to manage your diabetes? Can you describe such a situation? What happens in these situations? How do you feel in these situations? How do you deal with these situations? What do you need in these situations?

After patients had provided informed consent, the interviews were recorded, transcribed verbatim, and analyzed with MAXQDA software using framework analyses (41). In addition, a secondary analysis was performed on data from previous research (interviews and focus group discussions with people with diabetes from socioeconomically deprived neighborhoods). These interviews were held with Surinamese, Turkish, and Moroccan patients, as well as with patients from lower socioeconomic groups in general, and included topics similar to those used in our own interviews. Parts of these interviews were already coded in MAXQDA, which enabled us to incorporate the relevant codes into our own analyses; the remaining interviews were analyzed using framework analyses (21, 42–45).

##### Observations

The daily practice of a diabetes nurse working in a socioeconomically deprived neighborhood in The Hague was closely observed. Observations were also made during a 6-week intervention called “*Dealing with Diabetes*” that was organized for Turkish, Moroccan, and Dutch people with type 2 diabetes in socioeconomically deprived neighborhoods in Amsterdam. This took place by means of a participant, non-structured observation. One of our research group (Charlotte Vissenberg) observed all patient consultations with the diabetes nurse for 2 days from 0800 to 1700 hours in September 2008. The diabetes nurse saw (on average) 20 patients per day. The researcher sat in the nurse’s office (unobtrusively at the back) and was introduced to each patient as a colleague who would observe the consultations. All patients were asked if they had any questions regarding this observation; moreover, each patient was guaranteed his/her anonymity and was asked to provide informed consent. None of the patients had any questions and none refused participation. During the group-based intervention “*Dealing with Diabetes*,” the researcher (Charlotte Vissenberg) sat at the back of the room whilst the patients took part in the intervention.

During the observations, the researcher (Charlotte Vissenberg) wrote down everything that she saw and heard. After each consultation, she checked her findings with the diabetes nurse and the health promoter to ensure/optimize validity. These field notes were subsequently analyzed using thematic charting.

##### Analysis of Forums for People with Diabetes

Finally, we analyzed all the public content of forums held for people with diabetes. It appears that individuals (subjectively) report worse health and more often seek health information online, than individuals (subjectively) report good health. Furthermore, much information sought online is related to “sensitive” health topics that people prefer not to discuss with others (46). Kummervold et al. found that almost half of their respondents discussed personal problems online that they did not discuss with other people (47). Therefore, we analyzed all the public content of a forum organized for people with diabetes (48).

This forum was not specifically intended for patients from socioeconomically deprived neighborhoods and we doubted that these patients would participate in such a forum. However, we expected younger patients from our target population to participate, which might provide us with useful information as well. We selected www.diabetesforum.nl because it was organized by a professional organization (the National Diabetes Association), very active (lots of activity of members but also lots of new users) and was accessible to us.

Particular attention was paid to comments that indicated a lower socioeconomic background, e.g., related to educational level or profession, and to writing that included language that we recognized from our needs assessment phase.

We analyzed all public content from this forum from until April 2010. The content was analyzed using selective coding, focusing only on barriers/facilitators to self-management and the role of significant others (e.g., relatives and friends) in the performance of self-management. Additional analyses were performed using framework analysis (41).

## Results

We start this section with a description of the intervention lay out (step 4 of IM) followed by an explanation how we came to this specific intervention lay out (steps 1–3 of IM: the results of the needs assessment, performance and change objectives, the intervention methods and strategies).

### The Intervention Layout

This section describes the way the intervention was set up for the participants. *Powerful together with Diabetes* is a group intervention that lasted 10 months and consisted of the following 32 meetings: (i) 24 for participants (10 per group), (ii) 6 for their significant others, and (iii) 2 social network therapy sessions, which was attended by the participants and their significant others. These three components are described separately below.

#### Meetings for Participants

##### Phase 1

Phase 1 focused on providing the participants with the basic tools to manage their diabetes. During this phase that lasted 3 months, participants came together every week for 2 h in a community center (within walking distance from their homes) under the supervision of a group leader. During phase 1, five topic were discussed: what is diabetes (one meeting), blood glucose levels (two meetings), medications (two meetings), diet (four meetings), and exercise (two meetings). The last two meetings entailed a module of choice and the celebration of the end of phase 1. Each meeting centered around one topic and started with drinking coffee and tea followed by interactive games, quizzes, and role-playing exercises combined with energizers (fun exercises to stimulate bonding between participants and to provide a break for participants to increase the attention span). At the end of the meeting, the participants walked with each other around the neighborhood. Table 2 presents an overview of the topics.

**Table 2:** Overview of the topics in the meetings for participants: phase 1.

Meeting	Topic	Content of meeting
1	What is diabetes?	Getting to know each other (energizer) Glucose, insulin and the origin of diabetes (sugar disease game) Watching a DVD

2	**Blood glucose levels**	

2.1	Blood glucose levels	Review of the last meeting and exchange of experiences Collection of questions Information about high and low blood glucose levels (sugar disease game) Exchanging experiences and advice about recognizing and dealing with high/low blood glucose levels (letter of the week) Exchanging advice and practicing together how to deal with fear of getting a high/low blood glucose levels (letter of the week and role-playing) DVD Walking with group members

2.2	Monitoring of blood glucose levels (Meeting 1 for significant others)	Review of the last meeting and exchange of experiences Collection of questions Weighing the pros and cons of monitoring blood glucose levels because they might be too high (letter of the week) Practicing and exchanging advice together about monitoring of blood glucose levels in company (letter of the week and role-playing) Walking with group members

3	**Medications**	

3.1	Medications	Review of the last meeting and exchange of experiences Collection of questions Weighing the pros and cons of medication use (letter of the week) Exchanging experiences and advice about difficulties adhering to medication guidelines (role-playing) Exchanging experiences and advice about how to deal with forgetting medications (letter of the week) Information about medications (sugar disease game) Walking with group members

3.2	Medications	Review of the last meeting and exchange of experiences Visit from a diabetic nurse: opportunity to ask questions about own medications (participants brought own medications to the meeting) Exploring own medications with diabetic nurse: what are the different medications for? how to use these medications? DVD Information on influence of medications on blood glucose levels when exercising heavily, when ill, when forgetting medications (sugar disease game) Walking with group members

4	**Diet**	

4.1	Diet 1 (*Meeting 2 for significant others*)	Review of the last meeting and exchange of experiences Collection of questions Information about a healthy diet (nutrition game) Doing groceries (information on labels/explanation of logos) Walking with group members

4.2	Diet 2	Review of the last meeting and exchange of experiences Exchange of experiences and advice about barriers to eating healthy (letter of the week and role-playing) Walking with group members

4.3	Diet 3	Review of the last meeting and exchange of experiences Exchange of experiences and advice about resisting temptations (letter of the week) Practicing and exchanging advice together about resisting food in social situations (letter of the week and role-playing) Eating at regular intervals (group discussion) Walking with group members

4.4	Diet 4 (*Meeting 3 for significant others*)	Review of the last meeting and exchange of experiences Visit from a dietician: group members can choose between visiting a supermarket (how to pick healthy food from all the labels, how to read food labels), or adjusting their recipes to make them healthier Recipes: cookbook Walking with group members

5	**Exercise**	

5.1	Exercise 1	Review of the last meeting and exchange of experiences Weighing the pros and cons of physical activity How much do I exercise (group exercise)? How can we incorporate physical activity into our daily lives? Walking with group members

5.2	Exercise 2	Review of the last meeting and exchange of experiences Exchanging experiences and advice on how to deal with peer pressure not to exercise (letter of the week) Practicing and exchanging advice on strategies to overcome peer pressure regarding exercise (role-playing) Walking with group members

6	Module of choice	Review of the last meeting and exchange of experiences Participants can choose one of the following modules: diabetes on holiday, diabetes and Ramadan, smoking and/or sexual problems

7	Celebration: end of phase 1	Review of the last meeting and exchange of experiences Looking back at phase 1 Graduation phase 1: diploma

In phase 1, recurring program components were as follows: question time, sharing positive news, sugar disease game, nutrition game, letter of the week, role-playing, energizers, exercising, weighing pros and cons, homework, cookbook, summarizing results, and complimenting the participants. These components were adapted to the gender and cultural background of the different groups (e.g., letter of the week for the Surinamese patients could be about combining medications with nostrums; for Moroccan and Turkish women, it could focus on the fear of becoming addicted). Table 3 lists these program components.

**Table 3:** Program components: phase 1.

Program components	Description
Review last meeting and exchange of experiences (10–15 min)	At the start of each meeting, the group leader discusses how the period since the last meeting has been, and how the participants worked on their homework. Participants were stimulated to ask questions, exchange experiences, and help each other with their homework
Collection of questions (5–10 min)	To guarantee that the meeting fits the needs of the participants, the group leader starts with a short description of the meeting and writes down the participants’ questions on this topic. At the end of the meeting the group leader checks whether all questions have been answered
Sharing positive news (5 min)	To make participants more open for new information, they share positive news of the previous week with each other (self-affirmation). This news can cover any topic as long as it was experienced as positive by the participant
Sugar disease game (15–35 min)	Many meetings include a knowledge game. Participants participate in a quiz or a game, often teaming-up and competing with each other. The group leader only provides the information that participants ask for themselves. The aim is to only provide information needed by the participants and prevent giving an overload of information. The information provided was supported with visual aids from the Netherlands Institute for Health Promotion and Disease Prevention (NIGZ)
Nutrition game (60 min)	Participants were divided into groups and given plastic cards with photographs of dishes and foodstuffs. The cards are divided into breakfast, dinner, lunch, snacks, beverages and others. Participants can place cards on three different piles: green (eat as often as you like), orange (eat to a limited extent), and red (try to avoid, eat very rarely). They were asked to place each of these cards on the correct pile. Afterward, participants discussed the correct place for the cards with each other
Letter of the week (20–45 min)	This is a fictional letter from “someone with diabetes” who has a problem that needs to be solved. Participants are invited to brainstorm about the problem and help the writer of the letter to solve their problem. The letter of the week was used to uncover participants’ tacit views and provide them with solutions they might be able to use themselves
Role-playing 20 min (on average)	Every meeting included a role-playing exercise in which participants practiced together with some difficult situation. Participants could also provide role-playing scenarios themselves (e.g., difficult situations with which they were personally confronted). Each exercise ended with the exchange of advice and tricks/ideas the participants could use in their own lives
Energizers (5–15 min)	Energizers included passing a ball along and giving the person who fetches the ball compliments, balancing on a balloon to feel all the muscles in the body, playing “web of life” (a game that shows that everybody needs each other), keeping a balloon in the air, etc. The aim of these energizers is to stimulate bonding between group members and to refresh participants so that they are able to absorb new information again
Exercising with participants (30 min)	To show the participants how to exercise for 30 min and also let them experience this, each meeting the group leader walks with the participants for 30 min around the neighborhood (i.e., the participants’ own neighborhood). The group leaders are instructed to walk among the participants, so they can talk to everyone
Weighing pros and cons (15–20 min)	To change outcome expectations, the participants brainstorm about the pros and cons of certain behaviors (e.g., refusing food at a party). This can be done through group discussions, sometimes using a whiteboard to count the pros and cons. The group leader aimed to emphasize the pros to stimulate positive outcome expectations
Homework	The participants get homework at the end of each meeting. They were often asked to pay special attention to certain things (e.g., when do you smoke more than usual?) or to try and meet other participants outside of the meetings
Cookbook	The recipes of all participants were collected and compiled in a cookbook, which was given to the participants during the intervention. The cookbook also contained information about choosing healthy ready-to-eat meals for those participants who did not cook
Summarizing results and complimenting participants (10 min)	To help participants feel they had spent their time well, had helped each other and learned a lot, at the end of each meeting the group leader summarizes what the participants have learned, and tells participants that he/she is proud of them

##### Phase 2

Phase 2 focused on providing the participants with a set of (proactive) coping skills. Every meeting centered around one aspect of making an action plan: keeping a diary (two meetings), choosing a behavioral goal (one meeting), discussing problems and solutions (one meeting), identifying barriers and formulating solutions for these barriers (two meetings), practicing difficult situations (one meeting), discussing barriers and solutions and coping with these barriers (two meetings), making plans for the future (one meeting) and celebrating the end of the intervention (one meeting). Also in this phase, the meetings consisted of interactive games and role playing exercises alternated with energizers. At the end of the meeting, the participants walked with each other around the neighborhood.

In this phase, the meetings no longer took place every week. Gradually, more time was placed between each meeting thereby stimulating the participants to undertake activities together, without the group leader being present. The aim was to make participants more independent and to stimulate communication and exchange of social support/social influences outside of the regular meetings. The first two meetings were only 1 week apart; meetings 2–5 took place biweekly; meetings 6–9 took place once every 3 weeks and, finally, meeting 10 took place 4 weeks after meeting 9. Table 4 presents an overview of the topics included in these meetings.

**Table 4:** Overview of topics in meetings for participants: phase 2.

Meeting	Topic	Content of meeting
1	Diaries 1: physical activity, medications and blood glucose levels	Discussing the pros and cons of keeping a diary Keeping a diary for exercise, medications and blood glucose (group exercise) Walking with participants Meeting 4 for significant others
2	Diaries 2: nutrition and smoking	Review of the last meeting: exchange of experiences Comparing diary for exercise, medications and blood glucose with formal guidelines (group exercise) Keeping a diary for nutrition and smoking (group exercise) Walking with participants
3	Choosing a behavioral goal	Review of the last meeting: exchange of experiences Comparing diary for nutrition and smoking with formal guidelines (group exercise) Choosing a behavioral goal, action plan part 1 (group exercise) Walking with group members
4	Problems and solutions	Review of the last meeting: exchange of experiences Exploring barriers and thinking of solutions together, action plan part 2 (group exercise) Walking with group members
5	Barriers in the immediate social environments	Review of the last meeting: exchange of experiences Exploring barriers *in the immediate social environment*, action plan part 3 (group exercise) Special attention to feeling guilty about burden on significant others regarding the disease (letter of the week) Walking with group members Meeting 5 for significant others Social network therapy session 1
6	Solutions for barriers in the immediate social environment	Review of the last meeting: exchange of experiences Exploring solutions for barriers *in the immediate social environment*, action plan part 4 (group exercise) How to ask for help (letter of the week, brainstorming) Walking with group members
7	Practicing difficult situations	Review of the last meeting: exchange of experiences How to respond to peer pressure (role-playing) Asking for help, being assertive (role-playing) Walking with group members Homework: keeping diaries again
8	Barriers and solutions part 2	Review of the last meeting: exchange of experiences Comparing new diaries and old diaries, action plan 5 (group exercise) and discussing what goes well and what needs to be improved Walking with group members
9	Coping with difficult situations	Review of the last meeting: exchange of experiences Thinking ahead and being proactive in solutions Dealing with risky situations in the future, action plan 6 (group exercise) Walking with group members Meeting 6 for significant others
10	Plans for the future	Review of the last meeting: exchange of experiences What are we going to do in the future (group exercise) Dealing with risky situations in the future, action plan 7 (group exercise) Walking with group members Social network therapy session 2
11	End of the intervention	Review of the last meeting: exchange of experiences Looking back at phase 2 Graduation phase 2: diploma

Recurring program components were homework, review of the last meeting and exchange of experiences, keeping a diary, group exercises, making an action plan, energizers, and walking with other group members (Table 5).

**Table 5:** Program components: phase 2.

Program components	Description
Homework	In phase 2, the homework of the participants consisted of keeping their diaries, working on their behavioral goals and staying in contact with other participants in the weeks that had no intervention meeting

Keeping a diary	For this intervention, special diaries were developed for the participants to keep. They consisted of an outline of the days, which the participants could fill in. For filling in we used stickers, drawings or, if possible, writing. For example, for smoking we had stickers of little cigarettes, for physical activity stickers with a “10” on it (for 10 min) and stickers with different colors for the medications. The nutrition diaries could be filled in by means of writing or drawing

Group exercise (30–45 min)	The group exercises consisted of assignments the participants had to do in small groups (3 participants). Their aim was to let the participant practice and ask each other for feedback in a non-threatening environment, before sharing their experiences with the whole group. It also aimed to clarify what the participants were struggling with and to provide the group leader with guidelines for further explanations

Weighing pros and cons (15–20 min)	To change outcome expectations the participants brainstormed with each other about the pros and cons of certain behaviors (e.g., refusing food at a party). This was done through group discussions, sometimes using a whiteboard to count the pros and cons. The group leader aimed to emphasize the pros to stimulate positive outcome expectations

Action plan	The action plan consisted of 6 parts. It contained many pictures and consisted of outlines the participants had to fill in. Participants who could not write were teamed up with someone that could. The participants were not given all parts of the action plan at once, to prevent them from getting discouraged. They received a portfolio in which they added a part of their action plan each meeting; in this way they did not have to face all the work they still had to do, but could see their work growing Part 1: Choosing a behavioral goal, making it specific, determining who could help with this goal, and thinking of a reward when achieving this goal Part 2: Determining two important barriers to achieve the behavioral goal (some of the barriers were already listed in the action plan for the participants to mark). For each barrier, the participant has to create five solutions (together with group members) Part 3: Determining important barriers in the immediate social environment to achieve the behavioral goal and thinking of solutions Part 4: Thinking about ways significant others can help with diet, physical activity, taking medications, monitoring of blood glucose levels, and quitting smoking or smoking less Part 5: Updating action plan according to keeping a diary and comparing this diary with the one filled out in the beginning of phase 2 Part 6: Determining risky situations in the near future (the coming 2 weeks) and making plans to overcome these risky situations Part 7: Determining two new risky situations in the near future (the coming 2 weeks) and making plans to overcome these risky situations

Role-playing [20 min (on average)]	Every meeting contained a role-playing exercise in which the participants practiced together with a difficult situation from their action plan. The participants could also provide role-playing scenarios themselves (e.g., difficult situations they were confronted with themselves). Each exercise ended with the exchange of advice and tricks the participants could use in their own lives

Exercising with participants (30 min)	Continuing to walk for 30 min. Each time a different participant was responsible for the content of the exercise, or the route the participants took

Review of the last meeting and exchange of experiences, the energizers, and walking with group members are described in Table 2. In phase 2, some of the energizers focused on remembering information from phase 1 through games and/or exercises (e.g., throwing a ball and naming a green food from the nutritional game when catching the ball). Instead of the group leader initiating walking together and determining how long it would take and where they would go, in phase 2 the participants were encouraged to take the initiative. Participants had to choose what they would like to do (e.g., swimming instead of walking) to make them feel more independent and to make walking (with group members) easier.

#### Meetings for Significant Others of Participants

For the meetings with significant others, each participant was asked to invite two persons that they considered important for their DSM. In each phase, three meetings were held for significant others during which the diabetic patients were *not* present.

Phase 1 focused on increasing practical knowledge about diabetes and its treatment. It also aimed to make the significant others believe that self-management is necessary and to create awareness about their important role in this self-management. Phase 2 focused on supporting a relative or friend with managing diabetes. The aim was to make the significant others aware that self-management is a shared responsibility between themselves and the patient, and to make them feel confident to support the patient (self-efficacy and skills).

Generally, we used the same program components that were used in the group meetings for participants. The program components were also alternated with energizers. Other program components (Table 6) included letting the significant others experience certain behaviors themselves to help them empathize with the participant.

**Table 6:** Overview of the content of the meetings for significant others.

Meeting	Topic	Content of meeting
**Phase 1**		
1	Sugar disease and blood glucose levels	Getting to know each other (introduction of participants) Watching a DVD Glucose, insulin and the origin of diabetes (sugar disease game) Experiencing what it is like to measure blood glucose and thinking of ways to support someone with this (measuring blood glucose, followed by a group discussion) Homework ○ Reading the leaflet “What is diabetes?” ○ If they do not know how to use glucagon: ask a relative/friend or pharmacy
2	Medication and physical activity	Review of the last meeting: exchange of experiences Importance of taking medications and of physical activity (letter of the week, and weighing pros and cons) Brainstorming and exchanging advice about ways to support a relative/friend with taking medications and physical activity (group discussion) Homework: ○ To be physically active (e.g., by participating in Netherlands in Motion)
3	Healthy nutrition	Review of the last meeting: exchange of experiences Discussing the importance of healthy eating (weighing pros and cons) Information about a healthy diet (nutrition game) Brainstorming and exchanging advice about ways to support a relative/friend with eating healthy (group discussion)
**Phase 2**		
4	Diaries	Collection of questions Practicing filling in a diary and discussing ways to support someone with filling in a diary (group exercise)
5	Behavioral goals and improvement points	Review of the last meeting: exchange of experiences Collection of questions Choosing a behavioral goal (action plan: part 1) Thinking about helpful and non-helpful behavior (group exercise, weighing pros and cons)
6		Review of the last meeting: exchange of experiences Exchanging experiences and advice on how to help someone with diabetes regarding different topics: diet, medications, physical activity, smoking, monitoring of blood glucose levels (letter of the week) Module of choice (see Table 2)

#### Social Network Therapy Sessions (Participants and Their Significant Others)

In phase 2, both the participant and their significant others participated in two social network therapy sessions. Both sessions lasted about 25 min each and took place at the respondents’ home or, if preferred, at the community center.

During the first social network therapy session, the participant and their significant others determined a behavioral goal the participant could work on. This behavioral goal had to be based on the action plan used in the meetings for participants. Also, together with the group leader, they identified facilitators/barriers to achieve that goal and considered ways that the significant others could contribute to achieving this goal. The session ended with compiling a list of specific agreements that stipulated who will do what, and when, to achieve this goal.

The second social network therapy session evaluated the things that went well and the things that need to be improved. If necessary, a new/adapted list of agreements was complied.

#### Training and Supervision of Group Leaders

*Powerful Together with Diabetes* was delivered by various group leaders. The Turkish and Moroccan groups consisted of separate groups for men and women, whereas both men and women were included in the Surinamese group. Each group was guided by a group leader who was matched with the participants on ethnicity and gender. The leaders of the Dutch groups were diabetes nurses, GP assistants, and nurse practitioners, whereas the leaders of the Moroccan, Turkish, and Surinamese groups were migrant health workers.

All group leaders received 4 h of training before phase 1 and another 4 h before phase 2. In these training sessions, they participated in some of the intervention components themselves. They were also trained to use the handbook of phases 1 and 2, how to use the materials for the participants and how to guide and stimulate group bonding during the intervention. During these training sessions, they were provided with all intervention materials they needed during that phase. During the interventions, all group leaders had regular telephonic contact with the researchers; these calls helped with questions about the intervention and also provided practical advice. In this way, any problems were quickly and efficiently solved.

### Step 1: Needs Assessment and Development of the Logic Model

According to the IM method, we conducted a needs assessment to gain an understanding of the determinants underlying DSM and the target population of this study.

**Figure 1:**
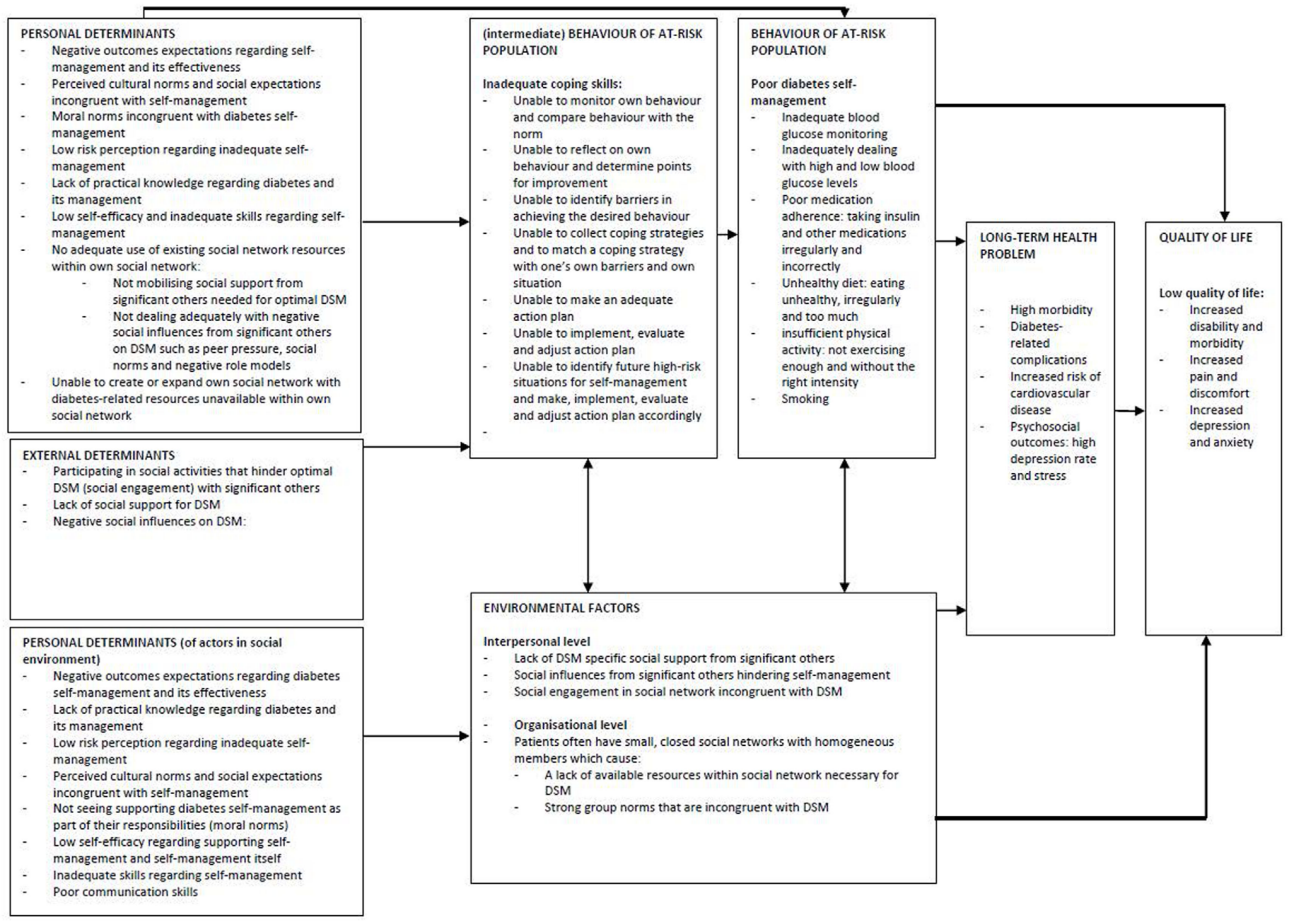
Results of the needs assessment: the logic model.

The results of the needs assessment are summarized in a logic model (Figure 1). A logic model describes the health problem, its impact on quality of life and its behavioral and environmental causes (35).

Our logic model is based on the results of the literature review and the qualitative study and outlines: (i) the personal and external determinants related to the self-management behaviors of the target population and (ii) also that of people in their immediate social environment. This section describes the development of the logic model.

#### Development of the Logic Model

**Figure 2:**
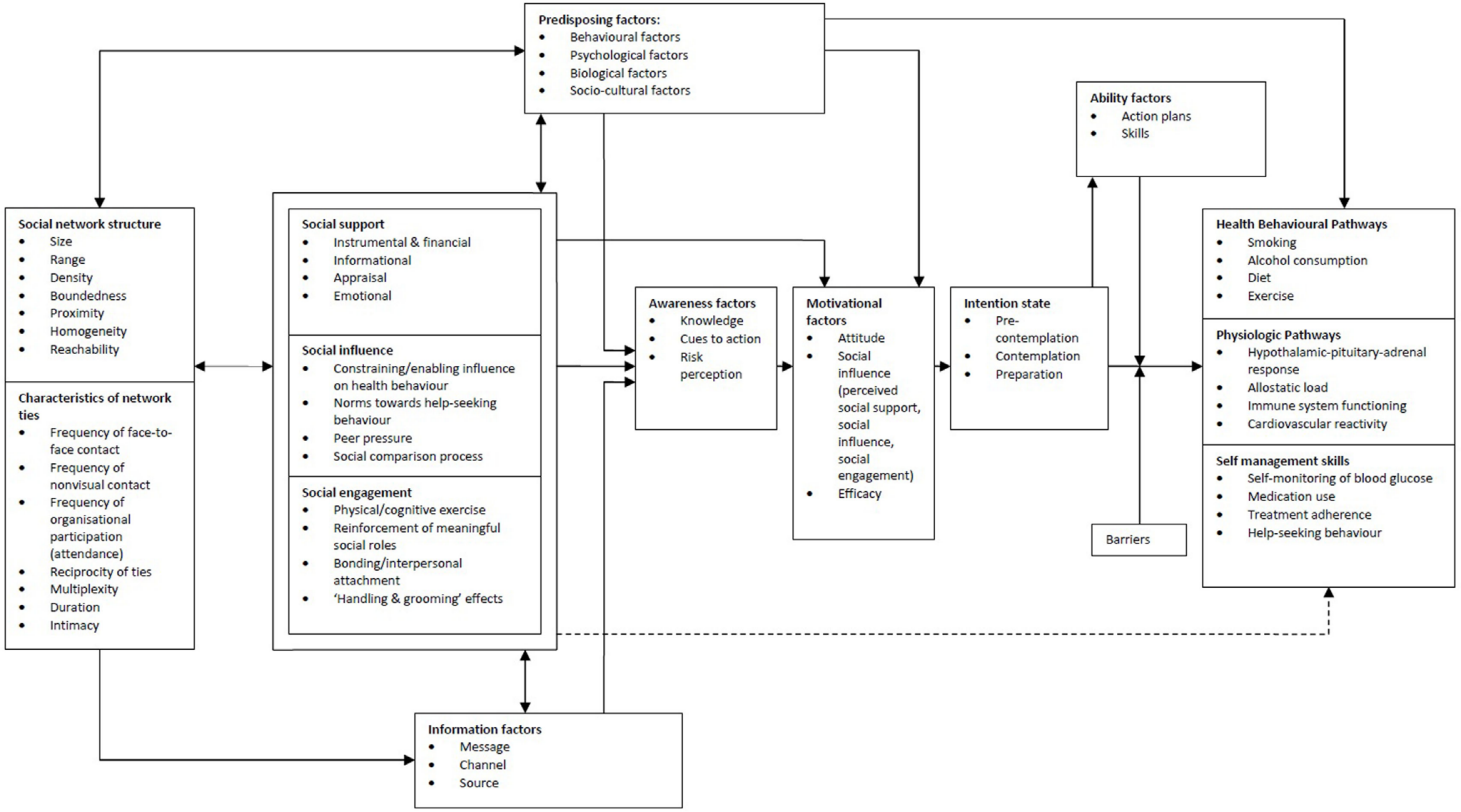
The I-Change model combined with the social network model of Berkman and Kawachi (50).

The needs assessment yielded many determinants related to the self-management of our target population. To explore these determinants, two theoretical models were applied that matched and further explained the results of the needs assessment, i.e., the I-Change model, and the transactional model of stress and coping (39, 49). The overall layout of the intervention is based on the I-Change model (49), which helped to decide in what order the determinants arising from the needs assessment should be addressed. However, because the needs assessment showed that the social network plays an important role in DSM, we combined the social network model of Berkman and Kawachi (50) with the I-Change model (Figure 2). Figure 2 shows the way we aimed for the intervention to address the determinants arising from our needs assessment. Our needs assessment showed that social influence might impact which health information reaches individuals (before awareness). We also observed that patients that received information, cues to action and/or were aware of their risks, were still hindered by social influences in their social environments (perceived social support, social influences, and social engagement) to change and maintain their behavior which is why social influences appears twice in Figure 2.

**Figure 3:**
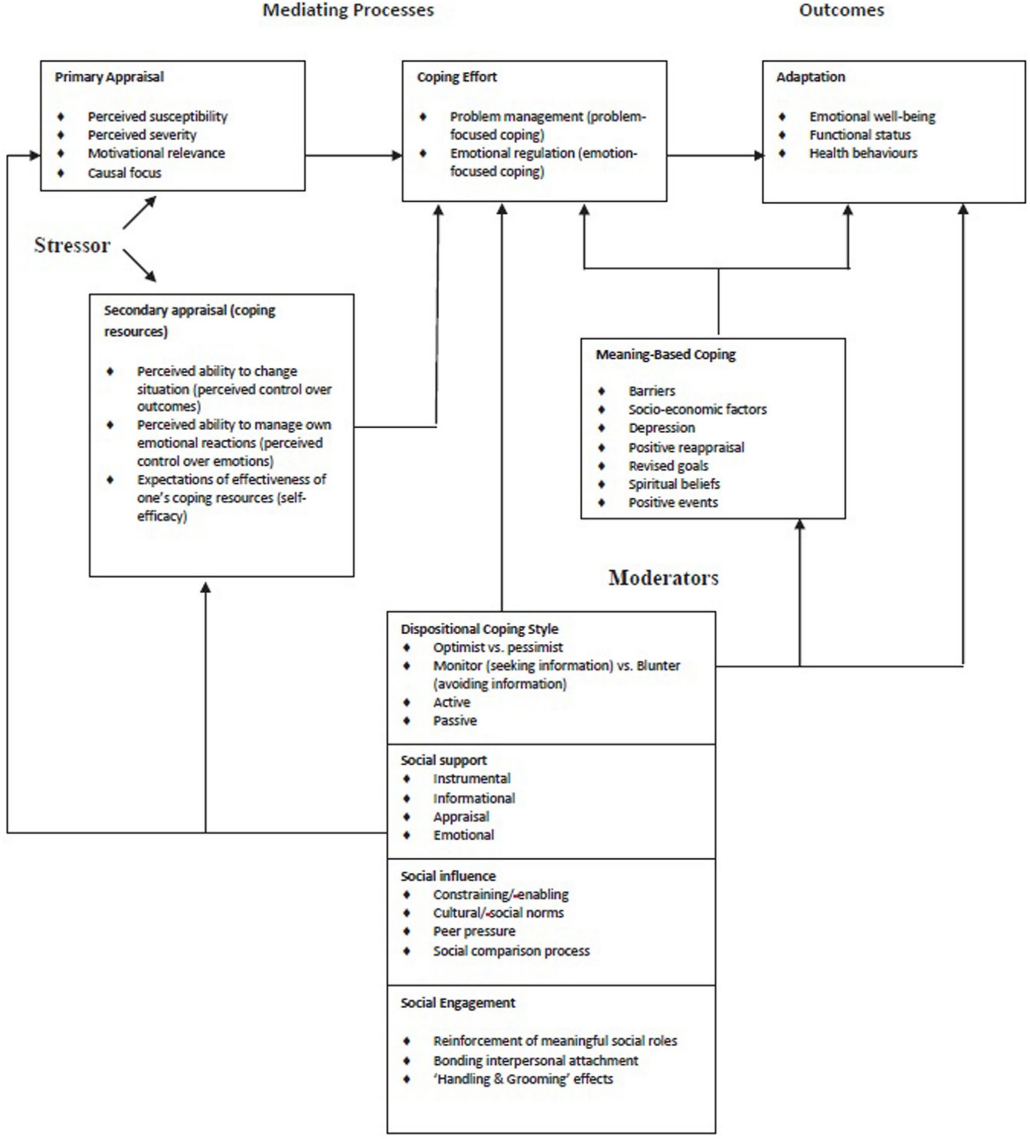
Transactional model of stress and coping combined with the social network model of Berkman and Kawachi (50).

The needs assessment also showed that our target population has inadequate coping skills; this represents a challenge for the implementation of self-management behaviors. This finding is in line with other reports on coping and socioeconomic position. Therefore, in our intervention, we decided to emphasize the items “Ability factors” and “Barriers” of the I-Change model. These were extended by replacing them with the transactional model of stress and coping. Again, because the needs assessment stressed the importance of the social network, we extended this model with the social network model of Berkman and Kawachi (Figure 3).

In short, the logic model assumes that our target population has a lower quality of life (increased disability/morbidity, increased pain/discomfort, and increased depression/anxiety) due to long-term health problems (high morbidity, diabetes-related complications, increased risk for cardiovascular disease, and negative psychosocial outcomes such as high depression rate/stress). These long-term health problems are related to poor self-management behaviors and ineffective coping skills caused by external determinants (social engagement/support/influences) and personal determinants (negative outcome expectations, perceived cultural norms and social expectations, moral norms, low risk perception, lack of practical knowledge, low self-efficacy, inadequate use of social network resources, and inability to expand social network with diabetes-related resources). It was also expected that the barriers (both external and personal) would be related to more distant determinants (such as unemployment, poverty, and marginalization) and other societal factors (in our original logic model) that might also influence long-term health problems. However, as Intervention Mapping stipulates focusing on the most important/changeable determinants, it was decided not to include these distant determinants but to consider them as the contextual environment in which the patients live. Although it is beyond the scope of this chapter to describe all the results of the needs assessment, the abovementioned determinants are in accordance with earlier studies on people with diabetes living in lower socioeconomic neighborhoods.

Therefore, here we describe only the most distinctive/new features of the intervention, i.e., specifically focusing on the social support/social influences affecting self-management, and on the educational requirements of these patients from a socioeconomically deprived neighborhood.

#### The Social Network and Self-management Behaviors

Diabetic patients that receive a lot of social support are better able to manage their diabetes than patients that receive little social support (22, 51–54). However, the immediate social environment of patients can hinder DSM. For example, negative interpersonal relationships (distrust, criticism, and dominance) also have a major impact on health and health behavior (23, 54–56). Non-supportive behaviors from family members and friends result in lower therapy adherence or lower metabolic control, and too much support (albeit intended by significant others) can be experienced as a form of interference (57–63). These social influences might have a stronger impact on health than social support alone (22, 50). There are indications that this is especially the case among people with lower health literacy (64). This stresses the need to examine the impact of all influences of the social networks of patients on self-management behaviors.

The social networks of individuals in socioeconomically deprived neighborhoods often consist of persons that are in the same situation as themselves. For example, in the Netherlands, low-income households are often clustered in the same neighborhoods and work environments (65). Moroccan, Turkish and Surinamese immigrants often live in neighborhoods and work in environments consisting mainly of people with the same non-Western ethnic background (66). For people in socioeconomically deprived neighborhoods, the neighborhood they live in and their family members generally form the most important source of their social contacts (65).

Social relationships between people that are in the same difficult situation (e.g., situations characterized by exclusion, stigmatization, and/or poverty) are often strong because of these shared experiences. The social networks they reside in are often smaller and less open than those of people with a higher socioeconomic status (26). The social networks of people in socioeconomically deprived neighborhoods often consist primarily of bonding social capital (social interactions between members of a homogeneous social network) and lack bridging social capital (social interactions that allow social network members to access resources other than those in their own social networks) (26, 65, 67).

According to the social network model of Berkman and Kawachi, the social network influences health through the following five mechanisms: (i) social support, (ii) social influence, (iii) social engagement, (iv) person-to-person contact, and (v) access to resources and material goods (50).

Social support consists of emotional, instrumental, informational and appraisal support. Social influence consists of constraining/enabling influence on health behavior, social norms, peer pressure, and social comparison processes. Social engagement consists of physical/cognitive exercise, reinforcement of meaningful social roles, bonding/interpersonal attachment, and “handling and grooming” effects. Moreover, the social network approach assumes that not every social network is necessarily beneficial for the health of its members. Also, some social networks are better in promoting health than others, but not everyone has equal access to these social networks (26).

Small, closed, and dense social networks, like those of our target population, might positively influence health because the exchange of social support is often high (26). However, the strong interdependence between social network members can also prevent members from acquiring new information and “getting ahead” in life (65, 67, 68). Small social networks are also associated with lower therapy adherence and lower metabolic control (59, 69). In addition, this type of social network is known to impose strong social norms on its members; when these social norms are incongruent with health behaviors, these social networks often have an adverse influence on the health of its members (70).

#### Social Network and Self-management Behaviors in Patients from Socioeconomically Deprived Neighborhoods

Five major themes related to the role of significant others in self-management behaviors emerged from the qualitative data of our needs assessment: (i) trying not to bother others, (ii) trying not to stand out at social events, (iii) peer pressure at social events, (iv) social norms regarding medication use and physical activity, and (v) having no “allies” in the immediate social environment.

##### Trying Not to Bother Others

Most respondents indicated that they considered their diabetes to be their own responsibility and did not want to bother their significant others (mostly partners, children and friends) with their condition. Most respondents did not see any advantage in asking for social support and indicated that they were capable of managing their diabetes by themselves.

As a result, these respondents often felt “bad” in front of their significant others if they had to take their diabetes into account. For example, some respondents said that they felt sorry for their partners because they always have to set the alarm clock to take their insulin on time, even when their partner wants to sleep. They often did their best not to burden their significant others with their self-management. As a result, our respondents (as well as the participants in the diabetes forum) indicated that they always have to be the stronger person, which demands a lot of self-control.

I often have to watch my husband eating a whole bowl of custard, cream and chocolate flakes. That’s really difficult, but I don’t want to put him under pressure – I’m the one who’s sick, not him … (Patient).

The health-care professionals indicated that relatives often know very little about diabetes and/or the ways to help out with self-management, or they think they are being helpful whereas this is in fact perceived differently by the diabetic patients.

Not asking for support also affected the ability of the respondents to make changes in their self-management. For example, all women from the ethnic minority groups indicated that it is difficult to cook something different, or serve more healthy food, when their significant others do not like it and/or refuse to eat it.

(…when serving brown rice instead of white rice): “Then they ask – what’ve you made now? Coconut?” (Patient).

##### Trying Not to Stand Out during Social Events

Most respondents were aware that they and their significant others shared the same unhealthy lifestyle. For most respondents, adhering to the diabetes guidelines meant behaving differently from their significant others. Most respondents did not want to draw attention to themselves and their diabetes, i.e., they did not want to stand out in general and especially not during social events.

Particularly the combination of not wanting to bother others and trying not to stand out during social events proved detrimental for their self-management behaviors. When eating at the house of a friend/relative, patients tried to blend in with the others and would not ask the host to take their diabetes into account (e.g., to eat at a certain time, or to make/buy special foods). This often caused uncomfortable situations (e.g., not eating everything that was served, or having to ask for something to eat before dinner) and often required extra self-management skills (e.g., eating in advance, rearranging their insulin dosages).

Social parties were also experienced as being difficult. The respondents did not want to bother the host with questions about the ingredients or ask him/her to make something especially for them. Therefore, they often did not know what they can/cannot eat. Also, it is difficult to predict how often people might come around offering snacks and what these snacks might contain. This combination of not asking for support and trying not to cause a fuss also made going out to dinner difficult. Respondents said they sometimes had to wait too long for their food and, because they do not always know what the ingredients are, this makes injecting the right amount of insulin a challenge.

Trying to “blend in” also affected their medication use. Most respondents did their best not to inject insulin during social situations. Some said that the looks from other people made them feel uncomfortable, others said that their relatives did not like to witness an injection (fear of needles), and sometimes asked annoying questions, or interfered too much.

##### Peer Pressure at Social Events

The respondents reported a lot of peer pressure and temptations at social events that affected their diet. Most said that they found it unpleasant when everybody was eating, whilst they either cannot or should not eat that particular food.

The Turkish, Moroccan, and Surinamese respondents indicated that the food plays a central role in their daily life. Offering food is seen as a sign of hospitality and it is customary to prepare extra food for guests. Refusing food is seen as impolite. Although respondents knew that they should not accept all the food that is offered, they did not want to hurt anybody’s feelings. Therefore, they often “act” as though they (temporarily) do not have diabetes, or try to avoid these situations as much as possible.

Almost all respondents had difficulty in resisting temptation and often felt under pressure to eat unhealthy/too much food, especially in the presence of negative role models.

“… when you’re at a party and other people with diabetes eat really unhealthy things”. Or ‘they’ (people at a party) say: “Well, this one and that one have got diabetes - and it’s OK for them” (Patient).

The respondents handle these situations differently: some accept that their blood glucose levels will be too low or too high.

##### Social Norms Regarding Medication Use and Physical Activity

The needs assessment showed that social norms were especially prevalent in medication use. All respondents indicated that they prefer not to take any medication at all. Medications are often regarded as “chemicals” that are not good for their body. Most respondents did not see their medication use as something permanent and hoped that 1 day they could live without medication. This was confirmed by the health-care professionals who reported that patients often think that if they lose weight they can live without medication. Most respondents had a strong aversion to insulin and indicated that they definitely did not want to use insulin in the future.

Then (when you have to use insulin) – that’s when you’re really sick (Patient).

According to the health-care professionals, Hindustani Surinamese persons often prefer not to take medications and have a strong tendency to see if they can manage without them. They fear that the medications will damage their kidneys; moreover, when they feel unwell they often skip their medication. Especially the use of insulin is experienced as a problem by these patients as it is associated with severe diabetes-related complications. We also observed that Surinamese patients sometimes try to “cleanse” their body by not using medications for a longer period of time. In addition, the interviews showed that Surinamese patients often get advice from other persons not to take their medications but to use “nostrums” (remedies from non-physicians) such as certain herbs or vegetables.

Turkish and Moroccan patients often have doubts about the medications prescribed by their physician. In Morocco and Turkey, physicians generally prescribe more medications and behave in a more authoritative way. Physicians in the Netherlands tend to ask more questions, which is interpreted by patients as lack of competance (3). The health-care professionals reported that, after the summer, these patients often arrive at consultations with a bag of (unnecessary) “new” medications they received from physicians in Morocco or Turkey. They also indicated that, among Moroccan and Turkish men, medications are sometimes associated with impotence.

During Ramadan, 60–80% of Turkish and Moroccan patients is non-adherent to their medications (71). Individuals who cannot participate during Ramadan due to illness are supposed to compensate by giving money to the poor. However, interviews with professionals revealed that this can be problematic when the individual involved has little/no money. According to the professionals, some alternatives, such as taking food to the poor, are also difficult because this is not socially accepted behavior in the Netherlands.

Social norms also affect physical activity. For example, Moroccan, and Turkish women mentioned they had no money to go to the gym, and that simply “walking around” was not an option for them. They were worried about what people in the neighborhood might think if they just “walked around” without going anywhere/without a valid reason. This situation was confirmed by the health-care professionals.

##### No “Allies” in the Immediate Social Environment

Most respondents said that they only knew a few people with diabetes and, often, they did not identify with them. For example, these acquaintances had different ways of dealing with their diabetes or were worse off than themselves, making it difficult to exchange ideas, ask questions, or share experiences. This affected multiple self-management domains. Also, quitting smoking was difficult because they were often surrounded by smokers and felt they were the only ones trying to quit.

… just try stopping when you’re living in a house with five smokers! (Diabetes forum).

Also, especially Dutch respondents said that they did not go to the gym (or go walking) because they had no one to go with, or had no one who thought it necessary to go to the gym.

#### Considerations Regarding Health Promotion in Patients from Socioeconomically Deprived Neighborhoods

The needs assessment also provided us with considerations related to health promotion in patients from socioeconomically deprived neighborhoods that needed to be taken into account when developing a group-based intervention for patients from socioeconomically deprived neighborhoods.

The respondents often had busy lives: they spend a lot of time taking care of their family (e.g., grandchildren and chronically ill relatives) and/or working. Some respondents felt stressed due to financial problems, or problems with raising their (teenage) children, or were worried about relatives living abroad. This was confirmed by the health-care professionals who reported that these patients often had difficult lives before the additional problem of developing diabetes.

You’re already having a tough time - then you also get one of the most difficult diseases that exist (Health-care professional).

Because of this, their disease was often given a low priority. Although they did what they had to do for their diabetes, most did not actively seek information about diabetes themselves. Some respondents expressed the desire for the diabetes nurse or the dietician to simply tell them what they had to do. According to the respondents, the diabetes regime is always complicated because one has to constantly think about the choices to be made and it is never simply “yes” or “no.” The health-care professionals stated that these patients do not necessarily want lots of medical information about their disease, but mainly want to know what they *have* to do and what they *cannot* do.

Second, the interviews revealed that most respondents have a low level of education, i.e., the majority had attained no, or only one, diploma. They had little learning experience, or their learning experiences were mostly negative, e.g., being unable to follow the classes, or being bored during lessons. These respondents were not convinced that “education” would help them to better understand their diabetes; they said that they were not suitable to learn things, or that “learning was not really their thing.” From our observations during the intervention “*Dealing with diabetes*” we knew that most participants had little experience with following classes. The more traditional educational methods (e.g., the teacher talks and the audience listens) did not seem suitable for this population, e.g., they had a short attention span and became distracted when they did not understand the information presented. Instead of asking questions, the participants generally chose to focus on something else (e.g., their telephones, or other participants).

Moreover, reading and writing was often a challenge for these respondents; this was confirmed by the interviews with the professionals. Also, the level of knowledge about diabetes differed between the respondents; some were unable to name one thing they had to do for their diabetes and could not recall whether they had ever heard of high/low blood glucose levels, whereas others could distinguish between their medications and also explain the basics of diabetes.

The needs assessment also revealed factors that might be important for the group process during the intervention. Most respondents were rather direct/blunt when we first met them (“rough diamonds”); however, this type of attitude can be problematic in a group where everybody needs to feel safe to speak freely. Moreover, it may be a challenge to find a balance between dealing with one’s problems in daily life whilst also focusing on the aims of the intervention.

#### Conclusions of the Needs Assessment

In conclusion, patients from socioeconomically deprived neighborhoods generally have social networks that seem less beneficial to self-management because of their small size and the limited ability to acquire new information. Furthermore, the strong social norms these social networks impose on their members seem incongruent with self-management behaviors. Moreover, these patients receive little social support for self-management behaviors because they often lack sources of support in their social networks and are reluctant to ask for social support or show others that they need it. These patients find it difficult to deal with influences from their social network such as various temptations, peer pressure, negative role models, and social norms. Moreover, for these patients, some of their significant others are unaware that they can/should help, or they simply do not know how to help.

During interventions for patients from socioeconomically deprived neighborhoods, the following aspects should be taken into account: low outcome expectations regarding education and low motivation for education; a low priority for diabetes; a desire for practical information; reading and writing difficulties; differences in knowledge about diabetes and factors that might affect the group process during the intervention.

### Step 2: Creating Performance and Change Objectives

The second step in IM is the development of matrices of change objectives that describe what needs to change in behavior and the environment to improve health and quality of life (35). We specified change objectives that describe what needs to change to achieve performance objectives, which in turn will lead to changes in behavioral and environmental conditions that will lead to accomplishing the program goals (35).

#### Formulating Program Goals

The needs assessment showed that our target population encountered influences that affected their self-management within their social networks (bonding social capital), including lack of social support, peer pressure, and social norms but also experienced difficulties in accessing other resources (bridging social capital) outside their social networks, such as new information, an ally/buddy, and other positive role models. In addition, they did not make adequate use of the social network resources already present in their social networks (asking for support, not dealing adequately with peer pressure, social influences and negative role models).

Therefore, based on the needs assessment and consultations with the experts, when we formulated program goals it was decided that our intervention should not only focus on the patient and their immediate social environment (bonding social capital) but also on bringing diabetic patients in contact with fellow patients (bridging social capital) thereby extending their social networks with diabetes-related resources. We aimed to extend the participants’ social networks with more diabetes-related resources while simultaneously making their own social networks more diabetes friendly. Accordingly, the following program goals were formulated for the social network that should be achieved by participation in the intervention:

(1) Extend the participants’ diabetes-related social networks, facilitating the exchange of social support and positive social influences with group members,
(2) Increase the participants’ ability to handle social influences that hinder their self-management such as norms, peer pressure, and temptations,
(3) Increase the engagement and support of the participants’ significant others in their self-management.

#### Performance and Change Objectives

This section focuses on the performance and change objectives that were formulated to achieve the program goals for the social network. The program goals for the social network were translated into performance objectives and change objectives. Table 7 provides an overview of the performance objectives for the entire intervention. The health-promoting behaviors of the social network are formulated as performance objectives but also as change objectives: objectives for determinants supportive of self-management behaviors.

**Table 7:** Performance and change objectives of the intervention *Powerful Together with Diabetes*.

Performance objectives	The participant	The participant in relation to their significant others	The significant others	The support group of the participants
Phase 1	1. Participant deals adequately with diabetes 1.1. Participants know the origins of diabetes 1.2. Participants know the basics about what happens in the body 2. Participant is therapy adherent with regard to medications 2.1. Participant takes his medications correctly and consistently every day 2.2. Participant takes his insulin correctly and consistently every day 3. Participant optimally manages his blood glucose levels 3.1. Participant self-monitors his blood glucose levels correctly and consistently 3.2. Participant adequately deals with high/low blood glucose levels 4. Participant has a healthy eating pattern 4.1. Participant eats sufficient healthy foods every day 4.2. Participant exercises regularly every day 5. Participant exercises enough 5.1. Participant exercises enough every day 5.2. Participant exercises with the right intensity every day 6. Participant does not smoke 6.1. Participant quits smoking	1. Participant tells significant others which obstacles he encounters with the correct and consistent intake of medications 2. Participant tells significant others which obstacles he encounters when managing blood glucose levels 2.1. Participant tells significant others which obstacles he encounters when monitoring blood glucose levels 2.2. Participant tells significant others which obstacles he encounters when dealing with high/low blood glucose levels 3. Participant tells significant others which obstacles he encounters when maintaining a healthy eating pattern 3.1. Participant tells significant others which obstacles he encounters when eating sufficient healthy foods 3.2. Participant tells significant others which obstacles he encounters when trying to eat regularly 3.3. Participant tells significant others which obstacles he encounters when trying not to eat too much each day 4. Participant tells significant others which obstacles he encounters when being physically active 4.1. Participant tells significant others which obstacles he encounters when exercising sufficiently 4.2. Participant tells significant others which obstacles he encounters when exercising with the right intensity 5. Participant tells significant others which obstacles he encounters when not smoking 5.1. Participant tells significant others which obstacles he encounters when trying to quit smoking	1. Significant others gather basic information on diabetes 1.1. Significant others attend the meetings that they are invited to attend 1.2. Significant others collect new information on diabetes 2. Significant others support the patient to take medications correctly and consistently 3. Significant others support the patient to take insulin correctly and consistently 4. Significant others support the patient to manage blood glucose levels 4.1. Significant others support the patient to correctly monitor blood glucose levels 4.2. Significant others support the patient to correctly deal with high/low blood glucose levels 5. Significant others support the patient to maintain a healthy eating pattern 5.1. Significant others support the patient to eat sufficient healthy foods 5.2. Significant others support the patient to eat regularly 5.3. Significant others support the patient to not eat too much 6. Significant others support the patient to be physically active 6.1. Significant others support the patient to exercise sufficiently 6.2. Significant others support the patient to exercise with the right intensity 7. Significant others support the patient to quit smoking	Participants in the support group continue to participate Participants in the support group experience the atmosphere as positive and pleasant Participants in the support group experience the meetings as fun and informative Participants in the support group trust each other and feel safe with each other Participants in the support group share experiences with each other Participants in the support group listen to each other Participants in the support group respect each other’s opinions

Phase 2	1. Participant monitors his own behavior (medications, insulin, nutrition, physical activity and smoking) 1.1. Participant compares his own behavior with the norm 1.2. Participant specifies goals for own behavior 2. Participant indicates obstacles to achieve goals 2.1. Participant indicates internal obstacles 2.2. Participant indicates obstacles in immediate social environment 3. Participant collects possible coping strategies to overcome obstacles when achieving goals 4. Participant chooses coping strategy that fits him and his problem(s) 5. Participant makes an action plan to implement the chosen coping strategy 6. Participant carries out the action plan 7. Participant evaluates the action plan and adjusts it when necessary 7.1. Participant evaluates the action plan 7.2. Participant adjusts the action plan when necessary 8. When experiencing a relapse, the participant interprets this positively (not as a failure) and goes back to 3 8.1. Participant interprets relapse positively 8.2. Participant goes back to 3	1. Participant tells his significant others what support he needs 2. Participant brainstorms with significant others about what needs to be changed to receive this support 2.1. Participant brainstorms with significant others about what he can change about himself 2.2. Participant brainstorms with significant others about what they can change about themselves 3. Participant makes agreements with significant others about giving and receiving support 4. Participant implements the appointments with significant others 5. Participant evaluates the implementation of the appointments with significant others 6. Participant adjusts the agreements together with significant others when necessary 7. Participant asks for support of significant others when experiencing a relapse and goes to 2 7.1. Participant asks for support after a relapse 7.2. Participant goes back to 2 after a relapse	1. Significant others see diabetes self-management (DSM) as a shared responsibility 2. Significant others regularly ask about how the DSM is going 2.1. Significant others regularly ask the participants how the DSM is going 2.2. When the DSM goes well the significant others give the patient compliments 2.3. When experiencing obstacles, the significant others give positive feedback 3. Significant others brainstorm with the participant about the source of these obstacles 3.1. Significant others brainstorm with the participant about obstacles within the participant 3.2. Significant others brainstorm with the participant about obstacles outside the participant 4. Significant others choose a constructive strategy to overcome these obstacles together with the participant 4.1. Significant others choose a suitable strategy with the participant 5. Significant others make agreements with the participant 5.1. Significant others concur with the participant on agreements about asking for and receiving support 6. Significant others keep these agreements 7. Significant others evaluate these agreements and adjust them when necessary 7.1. Significant others evaluate the agreements together with the participant 7.2. Significant others adjust the agreements with the participant when necessary and go to 2.1 8. Significant others avoid using punitive remarks when the participants experience a relapse 8.1. Significant others avoid punitive remarks 8.2. Significant others go to 2.3	Participants in the support group form a team Participants in the support group participate in activities together to improve their DSM (besides the regular group meetings) Participants in the support group keep on supporting each other with their DSM after the end of the intervention

	1. Participant identifies future risk situations for his DSM 2. Participant chooses the most suitable coping strategies to prevent these risk situations turning into a relapse 3. Participant makes an action plan 4. Participant implements the action plan before encountering high-risk situations 5. Participant evaluates his coping strategy and adjusts it when necessary 5.1. Participant evaluates his coping strategy 5.2. Participant adjusts the action plan when necessary and goes back to 4	Same as above	Same as above	

We organized two brainstorming sessions with five researchers who studied diabetes, nutrition, overweight and physical activity among patients in lower socioeconomic groups, or in minority groups. During these sessions, we checked the content of our performance objectives against their findings and experiences. Then, three researchers who had experience with Intervention Mapping, critically reviewed our performance and change objectives to see if they matched the Intervention Mapping conditions and were suitable to build our intervention on. Based on these meetings, our performance objectives were adjusted where necessary.

The performance and change objectives were formulated on the following four levels:

(1) the participant,
(2) the participant in relation to their significant others,
(3) the significant others,
(4) the participant’s support group as part of the intervention.

Tables 8–10 provide an example of the change objectives for the performance objective “Patient adequately monitors his/her blood glucose levels,” “Patient explains obstacles during monitoring of blood glucose levels to significant others,” and “Significant others support patient with adequate monitoring of blood glucose levels” (levels 1–3). Because we anticipated challenges in the group process of the intervention (which was a key aspect of this intervention) we also formulated performance and change objectives for level 4, i.e., the participant’s support group as part of the intervention (Table 11).

**Table 8:** Example of change objectives—the participant.

Performance objective	Personal determinants					External determinants	

	Attitude, outcome expectations	Perceived (cultural) norms and social expectations	Moral norms	Knowledge	Self-efficacy and skills	Social Support	Social influence
Participant monitors blood glucose levels correctly and consistently	Participant expects that monitoring his blood glucose levels correctly and consistently will provide more control and security Participant expects that he will understand his body better by monitoring blood glucose levels	Participant realizes that monitoring blood glucose levels is more important than trying to fulfill social expectations	Participant regards monitoring blood glucose levels correctly and consistently as a part of daily life	Participant knows why he needs to monitor blood glucose levels Participant knows why, how, and when he needs to monitor Participant knows that he has to monitor blood glucose levels before, during and after a day in the sun (holiday) Participant knows he has to monitor blood glucose levels more often during illness, or after a change in eating pattern	Participant feels confident that he can monitor blood glucose levels during social activities Participant feels confident that he can monitor blood glucose levels during special occasions Participant shows that he can attribute “bad” blood glucose levels as controllable Participant shows he can adequately deal with significant others who give him strange looks, or find it unpleasant when he monitors blood glucose levels	Significant others accept the monitoring of blood glucose levels by the participant Significant others indicate that the monitoring of blood glucose levels is necessary Significant others make sure the participant has a quiet place to monitor his blood glucose levels or Significant others find it normal that the participant monitors blood glucose levels in their company	Significant others accept that the participant regularly monitors blood glucose levels and support him

**Table 9:** Example of change objectives—the participant in relation to their significant others.

Performance objective	Personal determinant				External determinants	
	Attitude, outcome expectations	Perceived (cultural) norms and social expectations	Moral norms	Self-efficacy and skills	Social support	Social influence
2.1. Participant tells his significant others which obstacles he encounters when monitoring his blood glucose levels	Participant expects that informing his significant others will not affect his autonomy, but will make monitoring blood glucose levels correctly and consequently easier The participant expects that informing his significant others will enable them to better support him when monitoring his blood glucose levels The participant expects that the burden he will put on his significant others will be acceptable	Participant realizes that his significant others might influence the management of his blood glucose levels: – Asking annoying questions – Giving funny looks or being disgusted – Acceptance of monitoring – Not being helpful with high or low blood glucose levels Participant realizes that his significant others cannot take his needs into account if he does not inform them Participant realizes that informing his significant others is more important than blending in with the others (i.e., acting as though nothing is wrong; acting as normal as possible) Participant realizes that informing his significant others is more important than being afraid of burdening them	Participants regards informing his significant others as a part of his responsibilities	Participant is confident to dare and to be able to inform his significant others about the obstacles he encounters when monitoring his blood glucose levels Participant is able to show how to inform his significant others about the obstacles he encounters when monitoring his blood glucose levels	Participants in the support group help with thinking about the best ways to inform each other’s significant others about the obstacles they encounter when monitoring their blood glucose levels Significant others indicate that they are interested in the obstacles the participant encounters when monitoring his blood glucose levels	Participants in the support group encourage the participant to inform his significant others about the obstacles he encounters when monitoring his blood glucose levels Participants in the support group exchange positive experiences with informing significant others about the obstacles they encounter when monitoring his blood glucose levels Participants in the support group inform their significant others (positive example for other group members) about the obstacles they encounter when monitoring their blood glucose levels Significant others encourage the participant to tell them about the obstacles he encounters when monitoring his blood glucose levels

**Table 10:** Example of change objectives—the significant others.

Performance objective	Personal determinants				
	Attitude, outcome expectations	Knowledge	Perceived (cultural) norms and social expectations	Moral norms	Self-efficacy and skills
4.1. Significant others support the participant when monitoring his blood glucose levels correctly and consequently	Significant others expect that the participant will be better able to manage his diabetes when he regularly monitors his blood glucose levels Significant others are receptive to new information about the monitoring of blood glucose levels	Significant others know that the monitoring of blood glucose levels is an important part of DSM Significant others know how and when the participant needs to monitor his blood glucose levels Significant others know that the participant needs to monitor more often when the participant’s temperature is high, when the participant is ill, or when the participant alters his eating pattern	Significant others realize that the monitoring of blood glucose levels is more important than blending in with the others	Significant others regard supporting the participant with the monitoring of his blood glucose levels as part of their responsibilities	Significant others are confident that they can support the participant with the correct and consistent monitoring of his blood glucose levels Significant others can tell what kind of support the participant needs from them Significant others can tell how they can offer this support in the best way

**Table 11:** Example of change objectives—the participant’s support group.

Performance objective	Personal determinants				External determinants	
	Attitude, outcome expectations	Perceived (cultural) norms and social expectations	Moral norms	Self-efficacy and skills	Social support	Social influence
1. Participants in the support group continue to participate in the group meetings	Participants in the support group expect that participation will increase their control over their diabetes Participants in the support group expect that participation will motivate them to keep exercising, eat healthy and quit smoking	Participants in the support group believe that their group members will also keep on participating Participants in the support group feel that they are expected to keep on participating Participants in the support group realize that participating in the support group is more important than fulfilling other social expectations	Participants in the support group view participating in the support group as something that belongs to them Participants in the support group feel like part of the group	Participants in the support group are confident that they can keep participating in the support group Participants in the support group can show how to deal with negative remarks from significant others about participation Participants in the support group show how to conquer doubts and a lack of motivation to attend the group meetings	Participants in the support group support each other in dealing with negative remarks from significant others Participants in the support group support each other in dealing with doubts and a lack of motivation to attend the group meetings Participants in the support group give each other positive feedback when missing a meeting During each meeting, the group leader indicates how good it is that everybody is present Significant others facilitate the participant to attend the group meetings (taking care of the children, helping in the household, not nagging about the meetings, not prohibiting the participant to attend)	Participants in the support group keep participating (positive example for other group members) Significant others indicate that they appreciate that the participant keeps on participating

### Step 3: Selecting Theoretical Methods and Practical Strategies

In this step, we selected change methods based on the performance and change objectives. Based on these change methods we created practical strategies that formed parts of the program lay out (35). For this intervention, the practical strategies and program components were developed together with a psychologist who has considerable experience in working with lower socioeconomic groups. When selecting theory-informed intervention methods/practical strategies and producing program components/materials, the literature and other ongoing lifestyle interventions were scrutinized for methods and strategies that would be suitable for our target population. These practical strategies/program components were submitted twice to a panel of migrant health workers with a Turkish, Moroccan, and Surinamese background (*n* = 6). In addition, panel members were consulted individually about the specific cultural groups in our target population. Finally, some of the intervention components were pre-tested among the target population by means of focus group discussions (*n* = 3) in which we “practiced” some of the intervention components.

The next section describes the ways we used our needs assessment to choose methods and strategies for the intervention.

#### Methods and Strategies Specific for Patients from Socioeconomically Deprived Neighborhoods

This section describes the ways we considered the results of the needs assessment regarding our methods/strategies for our target population. From the needs assessment we knew that our target population had little (or primarily negative) experiences with education. Therefore, when selecting our theoretical methods we aimed to make learning as much fun and as interesting as possible. The aim was to make our participants curious about diabetes-related topics and make participation a positive experience by focusing on the abilities of our participants rather than on their shortcomings.

One of our strategies included an inductive educational approach (72). In contrast to deductive education that stems from theory, inductive education is built from the students’ experiences. Instead of telling students what they need to know from a theoretical point of view (deductive approach), we let the students practice with a problem they can relate to and slowly add information and theory to their understanding (inductive approach). Using this approach, the group leader can also investigate what the participants already know and which knowledge is incorrect or new (since the amount of foreknowledge about diabetes differed). Therefore, an inductive approach focuses on the abilities of the students and is closely connected with their interests (72). An example of the inductive approach is the game about nutrition: the participants had to solve a puzzle (what foods are green, which are orange and which are red?) together. The aim was to let them brainstorm together, focus on what they already knew, let them discover themselves what they did not know, and add to their knowledge and understanding where necessary.

To make learning as much fun as possible, it was important that the participants did not feel as though they were students but, nevertheless, felt that they benefited from each meeting. At the beginning and during the intervention, it was emphasized that they could help other participants with their own experiences and feedback (participatory problem solving). We did not use traditional educational strategies (such as teaching in front of a classroom) but non-traditional intervention strategies such as games and role-playing, with (fun or relaxing) energizers to optimize the attention span. The participants were encouraged to relate what they would “take home” from the intervention to help them realize what they had learned, or their particular significance for the other participants.

We also focused on self-affirmation by accentuating the personal qualities of the participants. This is a method to stimulate cognitive developments (72) and avoid dismissive/defensive reactions toward information perceived as a threat, and makes participants more perceptible for new information (73, 74). Practical strategies included giving each other compliments and constructive feedback, energizers, and sharing the positive news of the week at the start of each meeting.

To ensure a close connection to the interests of our participants, we involved them in the intervention through active learning to increase relevance and interest. This meant that the participants had direct influence on the topics and rehearsal situations addressed during the intervention.

Because most participants had a low educational background and problems with reading/writing, it can be difficult for them to learn/remember new information. Therefore, we used practical educational methods that enabled participants to remember the provided information and to practice real-life situations. Practical strategies included skills training with guided practice and feedback (practicing situations) and “chunking” (breaking up long pieces of information into easy to remember chunks). Also, a limited amount of information was provided at each meeting, and information from the previous meeting was always repeated at the latest meeting. In phase 2, the participants had to plan coping responses with the help of an action plan; this plan mainly consisted of the use of stickers and pictures.

Finally, to anticipate the varied and compelling priorities of our participants, the methods and strategies applied in this intervention focused on dealing with difficult situations that affected self-management; this was to promote/ensure long-term results. Therefore, the focus was on skills training with guided feedback. For example, participants exercised in their own neighborhood, and went to their own supermarket with a dietician to select healthy foods. Barriers that were encountered (e.g., an unsafe neighborhood, the higher costs of healthy food) were dealt with during these outings (e.g., exercising while shopping for groceries, finding alternatives that are also healthy, etc.).

#### Methods and Strategies for the Social Network

This section entails a description of the methods and strategies specific for the health-promoting behaviors of the social network. The needs assessment indicated that for interventions using group processes, it is important to consider how to shape these group processes for the participants. It is also important to consider how to balance dealing with personal problems and the goals of the intervention. Moreover, participants might have personal characteristics that can hinder the use of the group process during the intervention. Table 12 presents an overview of the methods and practical strategies for the health-promoting behaviors for the social network.

**Table 12:** Theoretical methods and practical strategies.

General objective	Subgoals	Theoretical methods	Practical strategies
1. Extending participants’ diabetes-related social networks, facilitating the exchange of social support and positive social influences with group members	– Participants positively influence each other (role models, positive peer pressure, positive group norms) – Participants encourage and support each other in adhering to their self-management during the intervention, and continue to support each other after the intervention has ended (advice, helping each other) – Participants continue to see each other after the intervention, and continue to do DSM-related activities together (e.g., exercising)	Skills training for providing and mobilizing social support Participatory problem solving Conscientisation methods Team building and human relations Stimulating communication and mobilizing social support	*Group meetings for people with diabetes: phases 1 and 2* – Participants took part in interactive games and energizers (short breaks during the intervention to keep the participants motivated and concentrated during the rest of the program: energizers often consisted of short exercises aimed at group bonding, e.g., throwing a balloon back and forth while giving each other compliments) in which they had to team-up with someone or form alliances. They were encouraged to open up to each other through these games and energizers – Participants were regularly invited to talk about their self-management problems and to ask group members for advice. To do this, the group members learned skills for giving constructive feedback – In small subgroups, participants did assignments in which they had to help each other (e.g., adjusting recipes together) to get used to giving and receiving social support – Participants had shared goals during the intervention such as making a cookbook together and attaining their diplomas – Participants were encouraged to phone and/or meet up with each other outside of the group meetings *Group meetings for people with diabetes: phase 2* – Periodic (first two weekly, then monthly) meetings were held. Participants were encouraged to continue seeing each other in between group meetings without the group leader

2. Increasing participants’ abilities to handle social influences that hinder their self-management, such as norms, peer pressure, and temptations	– Participants critically evaluate the impact significant others have on their DSM – Participants are better able to deal with social influences that hinder their self-management, such as peer pressure (e.g., pressure to eat unhealthy foods or to overeat, or negative feedback when exercising or taking medications)	Influencing normative beliefs by making peer expectations visible Building resistance to social pressure to engage in risk behavior Modeling and vicarious reinforcement	*Group meetings for people with diabetes: phase 1* – Group discussions were held about social situations in which managing diabetes is difficult (in response to a DVD, a letter of the week, and of their own accord) – Participant practiced these strategies with group members during role-playing exercises *Group meetings for people with diabetes: phase 2* – An action plan was drawn up in which social influences and dealing with social influences played an important part (group meetings). Together with other group members, the person with diabetes came up with strategies and solutions to overcome these difficulties

3. Increasing the engagement and support of the participants’ significant others in self-management	– Participants ask significant others for support – Participants indicate that their significant others are more involved in their self-management (providing more support or more enabling social influences) – Participants experience more enabling social influences – Participants experience fewer social influences from their significant others that hinder their self-management	Self-reevaluation Stimulating communication and mobilizing social support Modeling Participatory problem solving	*Group meetings for people with diabetes: phase 1* – Participants were encouraged to tell their significant others they have diabetes (if they did not know) – Participants were encouraged to tell their significant others about the negative social influences and barriers they face (social network therapy) *Social network therapy session: phase 2* – Participants discussed solutions and strategies with their significant others to deal with negative social influences on self-management – Together with their significant others, participants agreed on an action plan in which the significant others play an active role in their self-management. In this action plan, the participant and his/her significant other(s) described the problem they would be working on and barriers and facilitators to overcome this problem. Finally, they agreed on some concrete appointments with each other to overcome this problem *Group meetings for significant others: phases 1 and 2* – Significant others learned more about diabetes and the important role they play in the self-management of the patient with diabetes – To change their norms regarding self-management tasks, the significant others critically evaluated their own lifestyles through interactive games – Significant others did interactive assignments in which they distinguished helpful and non-helpful behavior with regard to self-management – Group discussions were held about ways to better facilitate the self-management of their relative with diabetes – Significant others learned ways to ask about their relative’s self-management in a friendly, supportive way (group meetings for significant others)

For this intervention to be successful, it was important that the participants became a mutual support group, i.e., support each other and positively influence each other in self-management behaviors. Therefore, during the intervention we focused on the group process and on establishing a safe learning environment, by increasing trust and the exchange of emotions/experiences between participants. The methods used to achieve this included team building and human relations, stimulating communication and mobilizing social support, and skills training for providing and mobilizing social support. Participants made agreements about trust and also agreed to treat the experiences/stories shared within the group in a confidential way. They participated in interactive games in which they had to team-up and form alliances. During the intervention, they established shared goals (e.g., making a cookbook together), were encouraged to share personal stories (e.g., by relating their positive news of the week), and the energizers were aimed at getting to know each other, having fun together, and appreciating each other (e.g., by giving each other compliments). Group members practiced giving constructive feedback and giving/receiving social support before implementing this in real-life situations.

The second goal was to increase the participants’ abilities to handle the social influences that hindered their self-management. Methods included the following: influencing normative beliefs by making peer expectations visible, building resistance to social pressure, modeling, and vicarious reinforcement. Practical strategies focused on making social influences on self-management visible by means of group discussions and stories about role models. Furthermore, strategies included helping fictional people with self-management problems, followed by giving advice to/asking advice from fellow group members. In phase 2, the participants made an action plan that focused on how to manage their diabetes within their social environments together with group members; in addition, they practiced the skills needed for implementing this action plan during role-playing exercises, followed by feedback from the group members and group leader.

Finally, the intervention aimed to increase the engagement and support of significant others in self-management behaviors. Methods to achieve this included self-reevaluation, stimulating communication and mobilizing social support, modeling, and participatory problem solving. Practical strategies included group meetings for significant others, in which the significant others learned the difference between supportive and non-supportive behaviors, communication skills, and how they might contribute toward self-management. Other strategies included the social network therapy sessions in which the patient and their significant others made an action plan together, which specified what each of them could do to achieve the joint goals.

## Discussion

*Powerful Together with Diabetes* primarily consists of non-traditional intervention strategies. The Intervention Mapping method focuses on matching theory and evidence based methods to the change objectives formulated in phase 2 of the intervention development and provides a state of the art overview of these methods (75). This helped our planning group to think out of the box, select the right methods and to create practical strategies that combined multiple methods at the same time.

Furthermore, the overviews of the change objectives and their matching methods and strategies facilitated our evaluation design. These overviews together with the data collected throughout the intervention period provided us with a thorough understanding of why certain aspects of the intervention worked while others did not.

However, lessons can be learned for future health promotion in this target population. First, though the intervention was appreciated and experienced as useful we noticed that the intervention did not fully fit the needs of the participants and seemed not totally in concordance with their daily lives. During the intervention, we realized we did not know the full extent of the problems those in our target population faced in their daily lives. Getting to know this target population takes time (76). Because of the nature and long time span of the intervention, we got to know the participants and their daily lives very well. Although the social network appeared to be a real problem for their DSM, this target population also faces other important problems. During the intervention, we noticed it did not fully meet the participants’ needs and did not seem entirely consistent with their daily lives.

Our participants often had multiple conditions, were experiencing financial problems, marital problems, domestic violence, or were caring for sick relatives. These problems had a major impact on DSM, and the priority they gave to DSM. Although the intervention aimed to teach participants to deal with these problems so they could self-manage their diabetes, this was not always realistic. For example, if your husband or son is abusing you, it is very unlikely he will become a supportive partner in your DSM.

Intervention Mapping stresses, the importance of conducting a needs assessment before developing the intervention. A health-related needs assessment includes a study of the determinants of behavior and environmental contributors to health problems or health risks (75). To do this, IM increasingly stresses the importance of participatory planning. Important elements of the needs assessment are the involvement of a planning group with planners, implementers, and program participants, and the involvement of the community throughout the whole project. Community involvement is needed to prevent a top-down, outsider approach (75, 77, 78).

IM thus advocates collaboration between community members and health professionals from the start of a project. Other researchers have also reported this to be a positive factor that helped in the development, adoption, and evaluation of an intervention (79–82). According to some studies, it can be difficult for health promoters to include the concerns and issues of the community because of the extra time needed for community involvement, and the often top-down organization of intervention development and top-down funding for these projects (78, 83).

When we started our research project, there was less emphases on participatory planning than there is now in the most recent version of the IM book (31, 35). Besides this, we also experienced the abovementioned limitations (a lack of time and top-down funding). When conducting our needs assessment, we performed all of the research activities as if we were developing a social support (and later on, a social network-based) intervention for patients with suboptimal glycemic control, as stipulated in our research grant proposal. This was the focus of the literature search, the interviews, the analyses of previously conducted interviews, and the diabetes forum. We did consult a panel of migrant health workers with Turkish, Moroccan, and Surinamese backgrounds multiple times. However, due to financial and time constraints, we did this fairly late in the process (after our needs assessment was completed) and asked for feedback only on topics related to our chosen focus, the social network.

Other factors complicated community involvement as well. We did not know the exact neighborhoods in which the intervention would be implemented, and so where our participants would live (which community to address). Also, we planned to aim the intervention at a very specific target population (with suboptimal glycemic control) and did not want to create false expectations within a community.

In hindsight, we conclude that the intervention could have been improved by investing more in participatory planning. If we had involved the target population and their community from the start and asked them (with no predetermined focus) what they thought would be the best when it comes to management of their diabetes, we might have come up with a different intervention, one with closer connections between the lives of our participants and the intervention (77, 83, 84).

In some of the interventions that report positively on community involvement, the researchers involved the community before applying for funding. They chose the study design and applied for funding together with the community or based on the results gathered together with the community (79, 82). However, as funding programs are often clustered around specific themes or have a predetermined focus (e.g., “preventing overweight by influencing lifestyle factors” or “socioeconomic health disparities, prevention and reduction through integrated local policies”) that provide a research direction, this might complicate involving communities without a predetermined vision (83, 85, 86).

In hindsight, it would have been better not to decide beforehand what the nature of the intervention would be, but to decide this based on an open needs assessment together with the target population and their community. Funding organizations might facilitate this community involvement by allowing for a longer planning period, and by allowing great flexibility in the area of focus and topics that will be investigated in one project (83, 87–89).

## Ethics Statement

This study was approved by the Medical Ethics Committee of the Academic Medical Centre (AMC) in Amsterdam. The participants provided written informed consent for the study and the study procedures.

## Author Contributions

CV coordinated the study, developed the intervention, constructed the design, and drafted the manuscript. VN and KS developed the study, constructed the design, and revised the manuscript. PU, BM, and GN participated in the design of the study and revised the manuscript.

## Conflict of Interest Statement

The authors declare that the research was conducted in the absence of any commercial or financial relationships that could be construed as a potential conflict of interest. The reviewer CH and handling editor declared their shared affiliation.
